# Using Medicare data to measure vertical integration of hospitals and physicians

**DOI:** 10.1007/s10742-020-00207-7

**Published:** 2020-02-04

**Authors:** Vivian Ho, Sasathorn Tapaneeyakul, Leanne Metcalfe, Lan Vu, Marah Short

**Affiliations:** 1 Baker Institute for Public Policy, Rice University, 6100 Main Street, MS 22, Houston, TX 77005 USA; 2 Department of Economics, Rice University, Houston, TX USA; 3 Department of Medicine, Baylor College of Medicine, Houston, TX USA; 4 Blue Cross Blue Shield of Texas, Richardson, TX USA

**Keywords:** Physician–hospital integration, Vertical integration, MD-PPAS, Validation, Physician practice ownership

## Abstract

Researchers, healthcare providers, and policy makers have become increasingly interested in the cost and quality effects of vertical integration (VI) between hospitals and physicians. However, tracking VI is often financially costly. Because the Medicare Data on Provider Practice and Specialty (MD-PPAS) annual dataset may be more cost-effective for researchers to access than private data sources, we examine the accuracy of MD-PPAS in identifying VI by comparing it to physician and hospital affiliations reported in Blue Cross Blue Shield Texas (BCBSTX) PPO claims data for 2014–2016. The BCBSTX data serve as a gold standard, because physician–hospital affiliations are based on the insurer’s provider contract information. We merged the two datasets using the physician National Provider Identifier (NPI), then determined what percentage of physicians had the same Tax Identification Number (TIN) in both sources, and whether the TIN implied the physician belonged to a physician- or hospital-owned practice. We found that 71.3% of successfully matched NPIs reported the same TIN, and 95.1% of patient-level observations were attributed to organizations with the same ownership type in both datasets, regardless of TIN. We compared regression estimates of patient-level annual spending on an indicator variable for physician versus hospital ownership for the primary attributed physician and found that estimates were within one percentage point whether one determined VI based on the BCBSTX or the MD-PPAS data. The results suggest that MD-PPAS, which costs less to obtain than from a for-profit data source, can be used to reliably track VI between hospitals and physicians.

**Electronic supplementary material:**

The online version of this article (10.1007/s10742-020-00207-7) contains supplementary material, which is available to authorized users.

## Introduction

Researchers, healthcare providers, and policy makers have become increasingly interested in the cost and quality effects of vertical integration between hospitals and physicians. Hospitals report their employment or other contractual relationships with physicians in the American Hospital Association (AHA) annual survey. The survey data has been used in studies to categorize hospitals by physician–hospital affiliation status based on their responses to the arrangements the hospital has with physicians (Baker et al. 2016; Ciliberto and Dranove 2006; Cuellar and Gertler 2006; Madison 2004; Scott et al. 2017; Short and Ho 2019). However, the AHA data do not provide information on which physicians each hospital has contractual relations with.

The company IQVIA maintains the SK&A database, which provides self-reported information on the owner(s) of the practices of office-based physicians. The database has been noted to include roughly 75% of active office-based physicians providing patient care in the American Medical Association Master file (Baker et al. 2016; Department of Health and Human Services 2012) and has been used in multiple recent vertical integration studies (Baker et al. 2016; Koch et al. 2017; Richards et al. 2016). However, IQVIA is a for-profit company, so the cost of obtaining their data is higher than from nonprofit and government sources. There is no set cost for the SK&A database. Researchers have been quoted prices of several thousand or tens of thousands of dollars, depending on factors such as the year(s) requested (i.e. previous years can be cheaper than the most recent year), the number of US States, and what physician identifiers or other variables are requested.

The Medicare Data on Provider Practice and Specialty (MD-PPAS) annual dataset may be a more cost-effective source for tracking vertical integration between hospitals and physicians. CMS charges $600 for each year of the MD-PPAS survey. The MD-PPAS reports the National Provider Identifier, the two most common tax identification numbers (TINs), and legal names used by each physician when filing claims with the Medicare program. The data has been used in previous analyses of physician markets, but not for studies of vertical integration (O’Malley et al. 2019; Studdert et al. 2019; Welch et al. 2014). While it is plausible that physicians with a hospital affiliation would submit both Medicare and private insurance claims under that same hospital, we know of no studies that have verified this behavior.

This study examines the accuracy of the MD-PPAS annual dataset in identifying vertical integration by comparing the identities of vertically integrated physicians and hospitals as reported in Blue Cross Blue Shield Texas (BCBSTX) PPO claims data for the year 2016 to affiliations recorded in MD-PPAS. The BCBSTX data serve as a gold standard, because physician–hospital affiliations for each claim are based on the insurer’s provider contract information.

## Methods

### The MD-PPAS data

The MD-PPAS data are research identifiable files that contains the NPI for each individual provider, the TIN used by each physician for billing, as well as the TIN’s legal name. In cases where a provider billed under more than one TIN in a given year, we used the primary TIN reported by MD-PPAS, which reflects the largest percentages of evaluation and management visits, procedures, imaging services, or non-laboratory tests with positive allowed charges amounts in the Medicare claims files (Centers for Medicare and Medicaid Services 2018).

Researchers can obtain the MD-PPAS by submitting a data request packet to ResDAC, the Research Data Assistance Center (Research Data Assistance Center 2019). ResDAC is a Centers for Medicare and Medicaid Services (CMS) contractor that provides free assistance to researchers interested in CMS data. The ResDAC submission requires a summary of the research plan, demonstration of Institutional Review Board (IRB) approval, as well as a description of confidentiality and security protocols to be implemented to protect the data. CMS currently charges researchers $600 for each year of the MD-PPAS that is requested.

### The BCBSTX claims data

The dataset we received from BCBSTX was based upon all preferred provider organization insurance claims processed for care through BCBSTX for 2014 through 2016 in the four largest Texas metropolitan statistical areas (MSA; Austin, Dallas, Houston, and San Antonio). The sample includes all claims for healthcare services, except for prescription drugs. We limited the analysis to adults ages 19–64 who were continuously enrolled in at least one of the calendar years 2014–2016.

We identified all claims from primary care physicians (PCP) and used these claims to attribute as many patients as possible to a PCP who was assumed to be responsible (directly or through referrals) for the majority of the patient’s care. The method for attribution is explained elsewhere in detail (Ho et al. 2019), and involves attributing each patient to the primary care physician they visited most in a 24-month window including each calendar year of data and the additional 12 months closest to it. Each provider submits their specialty when they request a provider record/credentialing with BCBSTX. However, BCBSTX designates physicians as PCPs in the claims data only if the insurer contracts with the physician to provide primary care.

For each patient, we summed all claims in each calendar year to calculate the annual allowed amount for each patient, which represents payments to providers by BCBSTX, as well as out-of-pocket expenses (deductibles and copayments) that the patient is responsible for. Annual spending for each patient represents all services used by patients, not just the services directly provided by the attributed physician organization. Patients with > $100,000 in costs in a calendar year were excluded from the sample, in order to exclude the effect of small numbers of very sick patients on average expenditures per patient (Ho et al. 2016; Robinson and Miller 2014).

Each insurance claim contains the NPI and TIN of the treating physician or hospital. The Network Management group at BCBSTX maintains records of the contracts negotiated with physician and hospital organizations, which also contain information on the identities of physicians included in each contract. This office supplied us with a list of each contracting entity and its associated TINs for the year 2016. The insurer knew which contracting entities submitted claims for hospital services, and any TINs listed in these contracts were defined as hospital-owned. Because we only receive the contracting list for 2016, we applied information from this year to all claims between 2014 and 2016.

The unit of observation in the BCBSTX data is a patient in a given year. Like the MD-PPAS, there are cases in which an NPI could be billing under more than one TIN. We determined the most common TIN associated with each NPI in the BCBSTX data when constructing descriptive statistics, but the TIN reported for each patient was maintained in the regression analyses.

### Merging and descriptive analysis

We merged the MD-PPAS and BCBSTX datasets by NPI. The merge created a dataset with annual spending for BCBSTX patients attributed to a primary care physician between 2014 and 2016 that contains the TIN reported in the MD-PPAS, as well as the TIN reported by the BCBSTX Networking Management group.

**Fig. 1:**
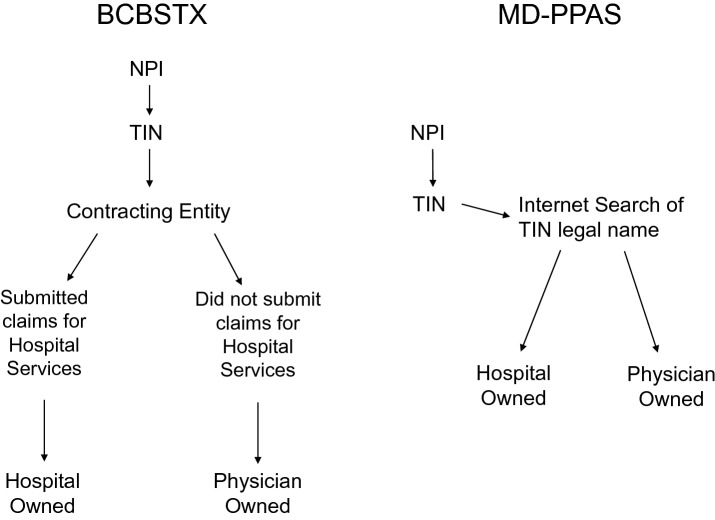
Attribution of tax identification numbers to hospital versus physician owned organizations

Figure 1 illustrates the methods used to distinguish TINs attributed to hospital versus physician owned organizations in the BCBSTX versus MD-PPAS datasets. The BCBSTX Network Management group classified each TIN as a physician or hospital organization. No such classification is available in the MD-PPAS. Therefore, for each merged NPI, we conducted an internet search of the TIN legal name reported in the MD-PPAS to determine whether the TIN belonged to a physician- or hospital-owned organization. For example, when the TIN legal name was the physician’s name, the practice was most likely physician-owned. However, there were a small number of cases where TIN legal names appeared to be for a physician practice, but the internet search revealed that the practice was in fact hospital-owned. In other cases TIN legal names that appeared to be for a hospital practice were instead found through the internet to be physician-owned.

The tightest form of vertical integration between physicians and hospitals occurs when physicians are subordinated as employees of a hospital (Robinson 1997). Some previous studies consider contractual relationships between physicians and hospitals as a looser form of vertical integration (Baker et al. 2014; Ciliberto and Dranove 2006). In this study, we only designated physician practices as vertically integrated with hospitals if our internet search revealed that the physicians were paid a salary by the hospital. We made this decision, because previous research has found that full integration of physicians as salaried hospital employees is associated with higher hospital prices and spending, while looser forms of integration display no such relationship (Baker et al. 2014). Co-author S. Tapaneeyakul performed the internet search of all TIN legal names to ascertain ownership type. Cases for which the internet search did not yield an immediately clear classification were reviewed and confirmed by co-author M. Short.

The MD-PPAS documentation states that 5.6% of NPIs had missing TIN names in 2016, and approximately 99% of TINs with missing names represent solo practices from 2013 to 2016 (Centers for Medicare and Medicaid Services 2018). That is, the TINs with missing names were associated with only one NPI in Medicare claims. Therefore, we assigned physician ownership to TINs without a reported legal name if the TIN was only associated with one NPI in the MD-PPAS database. A detailed description of the criteria used to classify each organization as physician- or hospital-owned is in Online Resource 1.

We first report the number of NPIs in the BCBSTX claims that were not reported in the MD-PPAS. Second, we report the percentage of NPIs for which both datasets report the same TIN, which is used for billing purposes. Third, we report the percentage of cases in which both datasets are in agreement on whether an NPI is associated with a physician- or hospital-owned organization. We further discuss patterns that we observe in discrepancies between the two datasets.

Fourth, we estimate a regression of annual spending for each BCBSTX member on the ownership type of the attributed PCP’s practice as determined by their TIN. This regression was estimated in a previous study which sought to compare annual health spending and quality of care for patients treated by doctors in hospital-owned versus physician-owned practices (Ho et al. 2019). We compare regression results using the ownership type as defined by BCBSTX versus a regression where ownership type is inferred based on MD-PPAS TINs and our internet search of TIN legal names.

The primary coefficient of interest is on the variable indicating whether the patient is being treated by a physician working in a hospital- versus physician-owned practice. We report this coefficient from the original specification and compare it to the result if the regression is estimated only for patients where the physician’s NPI was found in the MD-PPAS. In our previous study, we found that patients in BCBSTX preferred provider organizations incurred spending which was 5.8 percentage points higher when treated by doctors in hospital-owned versus physician-owned practices. Re-estimating this regression using only NPIs that we can match between the BCBSTX and MD-PPAS claims helps us assess whether the subsample of patients treated by NPIs that are also identified in the MD-PPAS is representative of the entire set of patients examined in our previous study.

For this subsample, we then re-estimate the regression using the classification of ownership type inferred from the MD-PPAS data combined with our internet search. We hypothesize that even though we are using vertical integration as defined by the MD-PPAS TINs and our internet search of TIN legal names instead of BCBSTX contract data, the estimated spending differential attributed to vertical integration will remain relatively unchanged. We also examine the sensitivity of these results by removing the assumption that patients are being treated at a physician-owned practice in cases where there was no legal name for the TIN number in the MD-PPAS database.

The other explanatory variables in the regressions include year fixed effects, as well as controls for practice size, age, gender, concurrent risk score, participation in a consumer directed health plan, MSA, and physician specialty. Definitions of these variables are in Online Resource 3. The Rice Institutional Review Board Chair approved the protocol under Exempt Review in accordance with Title 45, Part 46, Section 101 (b)4 of the Code of Federal Regulations.

## Results

There were 11,444 unique NPIs in the BCBSTX data. Table 1 contains summary statistics on the number of NPIs that we were able to identify in both the BCBSTX and MD-PPAS databases. The data merge yielded 8608 (75.2%) NPIs in the BCBSTX dataset that we were able to identify in the MD-PPAS. Of the 8608 matched NPIs, we were unable to assign ownership to two within the MD-PPAS database. Neither NPI reported a TIN legal name, which in most cases suggests that the corresponding TIN is associated with a physician in solo practice. However, both TINs were associated with multiple NPIs in the MD-PPAS dataset, and there was no way to determine whether these multi-member organizations were physician- or hospital-owned.

**Table 1:** Comparison of BCBSTX and MD-PPAS data by National Provider Identifier (NPI)

	Matching NPIs		Non-matching NPIs	
	Number	Percent	Number	Percent
*Sample*: *All BCBS NPIs* (*n* = 11,444)				
NPIs	8608	75.2	2836	24.8
Subset with 10 or fewer patients	3158	36.7	1985	70.0
Mean patients per NPI	164		43	
Mean patients per NPI per year	63		19	
Average expenditures per patient per NPI	$5973		$6144	

	Matching TIN		Non-matching TIN	
	Number	Percent	Number	Percent
*Sample*: *Matching NPIs with defined ownership type* (*n* = 8606)				
NPIs	6133	71.3	2473	28.7
Subset with 10 or fewer patients	1476	24.1	1680	67.9
Subset with matching ownership type	5810	94.7	1763	71.3
Mean patients per NPI	212		44	
Mean patients per NPI per year	79		18	

Among the 2836 unmatched NPIs, 70.0% had 10 or fewer patients attributed to them in the BCBSTX claims for the years 2014 through 2016. Only 36.7% of matched NPIs had 10 or fewer patients attributed to them in the BCBSTX claims. The unmatched NPIs treated an average of 19 patients per year, while the matched NPIs treated an average of 63 patients per physician per year. The unmatched NPIs had average expenditures of $6144 (CI $5876–$6412). In comparison, the matched NPIs had average expenditures of $5973 (CI $5819–$6126) in the BCBSTX claims.

For the 8606 NPIs that were successfully matched between the MD-PPAS and BCBSTX claims with ownership defined, 6133 (71.3%) had the same TIN that was most commonly used for billing. An additional 3.3% of NPIs had a secondary TIN in the MD-PPAS data that matched the most commonly used BCBSTX TIN. This finding may occur for a physician practicing at two facilities where one may be more likely to treat patients covered by the BCBSTX PPO, and the other may be more likely to treat Medicare patients. Physicians with matching NPIs across the two datasets but with different TINs tended to treat fewer patients than those with matching TINs. Of the 2473 physicians that had NPIs with non-matching primary TINs, 67.9% treated 10 or fewer patients, but of those with matching TINs only 24.1% treated 10 or fewer patients across the sample period. The physicians with matching NPIs across the two datasets and matching TINs in both datasets treated an average of 79 patients per year, while the NPIs with different TINs in the MD-PPAS versus BCBSTX data treated 18 patients per year on average.

Even though the TINs in the MD-PPAS do not match completely with the contract-based TINs reported in the BCBSTX claims, we sought to determine whether both datasets agree on the ownership type for the physician practice for each physician/NPI. Among the 8606 matched NPIs, 88% of NPIs had the same ownership type (physician vs. hospital owned) in both the MD-PPAS and BCBSTX datasets. There were 73 hospital-owned practices reported in the BCBSTX claims that we also identified in the MD-PPAS. Of these, we classified 20 as physician-owned in the MD-PPAS, because our internet search suggested that physicians in these practices were not salaried. These 20 practices treated 32,749 patients in the sample, which represents only 9.8% of all patients classified in the BCBSTX claims as being cared for by hospital-owned practices.

In the cases where both datasets had the same TIN for an NPI, the ownership type was also the same in 94.7% of cases. However, when the two datasets had non-matching TINs, the ownership type matched less often (71.3% of cases). Of the 8606 NPIs for which ownership was determined in both datasets, 1435 had a secondary TIN in the MD-PPAS database. In 221 cases, the primary NPI accounted for 60% or less of the total care provided by the NPI. Closer inspection of the secondary TIN in these cases determined that 52 (23.5%) of these NPIs were hybrids, meaning the primary and secondary TINs implied different ownership types.

Online Resource 2 provides a comparison of the distribution of patient characteristics by data sample and subsamples of matching versus non-matching NPIs, TINs, and ownership type. The distributions of patients in each cohort are similar across the board with the exception of city. The distribution of patients among the cities was similar for the full samples and the matching groups. But for the non-matching NPI, TIN, and ownership cohorts, there were shifts in the distributions. In particular, Dallas-Fort Worth had a disproportionately larger share of patients whose associated NPI was not matched between the two datasets while San Antonio and Houston had smaller proportions of patients with non-matching NPIs. Houston had a higher percentage of patients with non-matching TINs while Austin’s percentage of the total sample was smaller. And Austin had a disproportionately small number of patients with non-matching ownership type.

Table 2 contains results of regressions estimating the association between physician practice ownership type and annual patient spending using definitions of ownership type from either BCBSTX or the MD-PPAS data. The full regression results are represented in Online Resource 4. Column 1 contains the regression results using the full sample of BCBSTX claims with the insurers’ definition of practice ownership type obtained from their contracts. In this specification, annual spending per patient is 5.8% higher in hospital- versus physician-owned practices. Column 2 contains results for the sample of BCBSTX patients for which we could find an NPI in the MD-PPAS data. The sample size drops 7.9%, from 1,531,120 to 1,410,504. The higher spending differential for patients treated by hospital-owned practices changes from 5.8 to 6.1%, suggesting that the sample of patients treated by physicians with an NPI that was not found in the MD-PPAS is not markedly different from the full set of patients in the BCBSTX data.

**Table 2:** Adjusted effect of hospital versus physician ownership on median expenditures

	BCBS	BCBS (w/NPI in MD-PPAS)	MD-PPAS	MD-PPAS (missing TIN name ≠ physician-owned)	MD-PPAS (2016 only)
Hospital owned	0.0583**	0.0609**	0.0666***	0.0678***	0.0678***
	(0.0212)	(0.0190)	(0.0181)	(0.0181)	(0.0184)
	(0.0167–0.0998)	(0.0237–0.0980)	(0.0312–0.102)	(0.0323–0.103)	(0.0317–0.104)
Observations	1,531,120	1,410,504	1,365,477	1,329,200	443,366

Robust SE in parentheses and CI in brackets

The estimates are adjusted for practice size, year, age, gender, concurrent risk score, participation in a consumer-directed health plan, MSA, and physician specialty. The year indicator variables capture increases in spending resulting from inflation

***p < 0.001; **p < 0.01; *p < 0.05

Column 3 contains estimates using practice ownership type obtained from the MD-PPAS. The sample drops another 3.2% to 1,365,477, because ownership type and physician specialty could not be determined definitively using information in the MD-PPAS. Nevertheless, the estimated higher spending differential for patients treated by hospital-owned practices changes only slightly to 6.7%. If we do not assume that the 36,277 patient observations associated with an NPI that are missing a TIN name are physician-owned, removing these observations from the regression sample yields a spending differential of 6.8% in Column 4. As a sensitivity analysis, we re-estimated the specification reported in Column 3 using only observations from 2016, the year for which BCBSTX provided network contracting data. The spending differential again rose only slightly to 6.8% (See Column 5). We also tested whether the higher spending for hospital-owned practices is similar in the BCBSTX versus MD-PPAS datasets if one narrows the analysis to family medicine or internal medicine physicians, which are the two largest primary care specialties in our sample. The results are in Online Resource 5. For family medicine physicians, which account for roughly 70 percent of PCP claims in each dataset, the spending differentials are relatively similar; 5.5% in the BCBSTX claims and 5.2% if one determines physician–hospital integration and physician subspecialty using the MD-PPAS dataset. For internal medicine physicians, which account for slightly less than 30% of claims in both cases, the spending differential for patients in hospital-owned practices is 6.7% based on BCBSTX integration and subspecialty definitions. Using MD-PPAS integration and subspecialty definitions, the spending differential increases to 9.9%. Therefore, the MD-PPAS may not be reliable for measuring the spending effects of integration for subspecialties.”

## Discussion

The results suggest that the MD-PPAS dataset, which costs less to obtain than SK&A data, can be used to reliably track vertical integration between hospitals and physicians. In a private insurance claims database that includes NPIs, we determined that 75.2% of these NPIs could be matched to the MD-PPAS. Of these 8606 physicians we identified in both the BCBSTX claims and the MD-PPAS, 88.0% had the same ownership type in both datasets. This relatively high number suggests that the MD-PPAS dataset, which costs less to obtain than the SK&A data, can be used to reliably track vertical integration between hospitals and physicians.

We were unable to find 24.8% of NPIs listed in the BCBSTX claims in the MD-PPAS. Some physicians filing claims with BCBSTX did not treat Medicare patients and therefore are not included in the MD-PPAS database. For example, 1340 of the 11,444 NPIs in the BCBSTX sample were classified as pediatricians, and over 90% of them were not found in the MD-PPAS database. Furthermore, according to a recent survey, roughly 7% of primary care physicians were not accepting Medicare patients in 2015 (Boccuti et al. 2015). The set of 8606 NPIs found in both the BCBSTX and the MD-PPAS databases is the most important for studying vertical integration, because physicians who accept Medicare coverage treat the overwhelming majority of adult patients in the US. These physicians also face the greatest incentive to vertically integrate, because Medicare reimburses office visits more generously if they are billed under the hospital outpatient schedule versus the physician reimbursement schedule. This paper contributes to the literature by showing that vertical integration identified in the MD-PPAS data applies to privately insured patients treated by the same physicians. Furthermore, the unmatched NPIs tended to be for physicians treating fewer patients, which are likely to represent smaller physician practices. Thus, missing NPIs only required us to delete fewer than 10% of patients from our regression sample.

If one uses the MD-PPAS data to study physician–hospital integration, one must perform an extensive search of other sources to definitively determine which TINs are associated with physician versus hospital owned organizations. One must conduct ownership searches yearly, because some physician practices are forming or dissolving contractual relationships with hospitals in any given year (Short et al. 2017). We had the resources to conduct this search for 3 years of practice data in Texas, which is the third largest state in the US population. The data use agreement we signed to access the MD-PPAS bars us from sharing this information with other researchers. It would be helpful to identify a way for approved researchers to share information on vertical integration, to prevent duplicate performance of the same task.

While we were able to determine in the MD-PPAS data that a small number of NPIs practiced at both hospital and physician owned practices, we limited NPIs to the ownership of the primary TIN. Physicians practicing under multiple ownership arrangements may act differently than those under a single owner. But for our validation purposes, we were not able to take advantage of MD-PPAS’s ability to determine hybrid ownership since there was no comparable measure in the BCBSTX data.

The market and regulatory forces that determine integration between hospitals and physicians are drawing increased interest from policy makers and researchers. Studying this phenomenon requires access to comprehensive data on physician membership that can be readily linked to data on the quality of care and costs. The MD-PPAS can serve as a useful tool in this endeavor that can also lower the costs of conducting research relative to privately sold databases.

## Electronic supplementary material

Below is the link to the electronic supplementary material.

Supplementary material 1 (DOCX 33 kb)
