# Supplementary material for: Using Medicare data to measure vertical integration of hospitals and physicians
Source: Health Serv Outcomes Res Methodol. 2020 Feb 4;20(1):1–12. doi: 10.1007/s10742-020-00207-7 (PMC7036068; doi:10.1007/s10742-020-00207-7)
Supplement: Supplementary file 1 — Supplementary material 1 (DOCX 33 kb) [file 10742_2020_207_MOESM1_ESM.docx]

**Health Services and Outcomes Research Methodology**

**Using Medicare Data to Measure Vertical Integration of Hospitals and Physicians**

Authors:

Vivian Ho, Ph.D.

Chair in Health Economics, Baker Institute for Public Policy at Rice University; professor, Department of Economics, Rice University; professor, Department of Medicine, Baylor College of Medicine, Houston, TX, USA

[vho@rice.edu](mailto:vho@rice.edu)

Sasathorn Tapaneeyakul, Ph.D.

Leanne Metcalfe, Ph.D.

Lan Vu, BS

Marah Short, MS

**Online Resource 1.**

**Using MD-PPAS TIN Legal Names to identify Physician- versus Hospital-Owned Organizations**

For each NPI that appeared that was present in both the MD-PPAS and BCBSTX datasets, we conducted an internet search of the TIN legal name reported in the MD-PPAS to determine whether the TIN belonged to a physician- or hospital-owned organization. Following is a description of our methodology.

1. **For TIN legal names that appeared to be a physician’s name,** we searched for a website with information about the physician’s practice. Certain practices would indicate that they were independently-owned and operated. Some websites would list the name(s) of a hospital as a partnership or an affiliation, but the practice was clearly not owned by the hospital and therefore defined as physician-owned. A small number of websites explicitly stated that the practice was hospital-owned, along with the website hosted by the hospital and links to the main hospital website. These practices were classified as hospital-owned.

If no website containing the physician’s name was found, then we searched various databases such as NPIdb, the NPPES NPI Registry, HIPAASpace, or even LinkedIn for the physician’s name. These sources would list the physician’s workplace, sometimes with a timeline, that was used to determine ownership status. If one of these data sources listed the physician’s practice with no trace to a hospital, then the TIN was defined as physician-owned.

2. **For TIN legal names that appeared to belong to a physician group,** we searched for a dedicated website with that name. Similar to the approach used for TINs with a physician name, we used detailed information in the website to determine whether a practice was physician- or hospital-owned. Most were determined to be physician-owned. Sometimes a TIN name had a hospital name as part of their group name, or was listed within a hospital website. The classification decision would then be based on the description of the group within the hospital’s website. If this information was insufficient to make a decision, then the career page of the hospital that lists positions for physicians usually provided further detail about the position, group affiliation, salary, and how much freedom a physician would have within the specific position and group. Documents that contained an organizational chart or other information on the structure, legal activities (e.g. court case), and financial disclosure also helped with the decision when the aforementioned information was not sufficient. If there was no information related to a hospital in our searches, then the practice was assumed to by physician-owned.

3. **For TIN legal names that represented hospitals,** we consulted the hospital’s main web page using both the TIN information and provider’s name. The main page would contain a link to a physician’s page or a search option that listed information about a physician and sometimes whether the individual was employed at the hospital or belonged to a physician group. A career page as well as additional documents mentioned in item number 2 above were also used. The majority of these TINs were found to be hospital-owned.

4. **For TIN legal names that were indeterminate,** i.e. the legal name did not clearly indicate whether the entity was a hospital, medical group, or physician’s practice (e.g. non-hospital or -physician name or name ending with “clinic” or “primary care”): any information from the organization’s websites and other sources as mentioned previously would be used to decide on the status. There were also small number of cases in which the practices, groups, or names were not found or directly matched in the internet search. In these cases, the physician’s name associated with the TIN was used to determine the practice location. A combination of all information sources mentioned above were then used to decide on the organization status.

5. **For TINs with missing legal names for which ownership was not determined by any above means,** if the TIN was not used by any other NPI in the MD-PPAS data then based on the MD_PPAS documentation the TIN was defined as a solo practice and was assigned physician ownership.

| **Online Resource 2.  Distribution of Patient Characteristics by Data Sample** | | | |  |  |  |  |  |
| --- | --- | --- | --- | --- | --- | --- | --- | --- |
|  | **BCBSTX Data** | | | **BCBSTX and MD-PPAS Data Matched by NPI and Ownership Type Defined** | | | | |
|  | Full Sample | Matching NPIs | Non-Matching NPIs | Full sample | Matching TIN | Non-Matching TIN | Matching Ownership Type | Non-Matching Ownership Type |
| N | 1,531,120 | 1,410,504 | 120,616 | 1,410,500 | 1,237,215 | 173,285 | 1,340,699 | 69,801 |
| NPIs | 11,444 | 8,608 | 2,836 | 8,606 | 6,133 | 2,473 | 7,578 | 1,028 |
| Mean allowed expenditures/patient | $4,661 | $4,682 | $4,415 | $4,682 | $4,576 | $5,437 | $4,659 | $5,121 |
|  |  |  |  |  |  |  |  |  |
|  | *Percent Distributions* | | | | | | | |
| Age |  |  |  |  |  |  |  |  |
| 19-29 | 16.3 | 15.3 | 27.5 | 15.3 | 15.0 | 17.7 | 15.1 | 18.2 |
| 30-39 | 21.0 | 21.0 | 21.2 | 21.0 | 20.6 | 23.9 | 20.9 | 22.7 |
| 40-54 | 39.4 | 39.9 | 34.3 | 39.9 | 40.1 | 37.8 | 40.0 | 37.4 |
| 55-64 | 23.3 | 23.8 | 17.0 | 23.8 | 24.3 | 20.5 | 23.9 | 21.7 |
| Male | 44.6 | 44.8 | 42.5 | 44.8 | 46.3 | 34.3 | 45.1 | 38.5 |
| Consumer Driven Health Plan | 18.5 | 18.4 | 19.9 | 18.4 | 18.5 | 17.7 | 18.4 | 18.8 |
| City |  |  |  |  |  |  |  |  |
| Houston | 35.8 | 36.8 | 24.1 | 36.8 | 35.5 | 45.5 | 36.8 | 36.2 |
| San Antonio | 11.7 | 12.2 | 5.9 | 12.2 | 12.2 | 11.8 | 12.2 | 10.6 |
| Dallas-Fort Worth | 40.3 | 38.8 | 57.8 | 38.8 | 39.5 | 34.0 | 38.2 | 49.3 |
| Austin | 12.3 | 12.3 | 12.2 | 12.3 | 12.8 | 8.7 | 12.7 | 3.8 |
| Risk Score |  |  |  |  |  |  |  |  |
| Decile 1 | 7.9 | 7.7 | 9.9 | 7.7 | 8.0 | 6.0 | 7.7 | 7.6 |
| Decile 2 | 9.8 | 9.8 | 10.5 | 9.8 | 9.9 | 8.9 | 9.8 | 9.6 |
| Decile 3 | 10.1 | 10.1 | 10.4 | 10.1 | 10.2 | 9.3 | 10.1 | 9.7 |
| Decile 4 | 10.3 | 10.3 | 10.5 | 10.3 | 10.4 | 9.6 | 10.3 | 10.0 |
| Decile 5 | 10.4 | 10.4 | 10.2 | 10.4 | 10.5 | 9.9 | 10.5 | 9.9 |
| Decile 6 | 10.5 | 10.5 | 10.2 | 10.5 | 10.5 | 10.2 | 10.5 | 10.0 |
| Decile 7 | 10.4 | 10.4 | 9.8 | 10.4 | 10.4 | 10.7 | 10.4 | 10.4 |
| Decile 8 | 10.4 | 10.4 | 9.7 | 10.4 | 10.3 | 11.3 | 10.4 | 10.7 |
| Decile 9 | 10.1 | 10.2 | 9.6 | 10.2 | 10.0 | 11.8 | 10.1 | 11.0 |
| Decile 10 | 10.1 | 10.2 | 9.2 | 10.2 | 9.9 | 12.3 | 10.1 | 11.1 |

**Online Resource 3.**

**Variable Definitions**

| Consumer Directed Health Plan (CDHP) | Indicator variable=1 if the patient was in a consumer directed health plan; A CDHP combines a high deductible health plan (HDHP) as defined by the Internal Revenue Service (IRS) with a health savings account or a health reimbursement account. Patients with CDHPs may be less likely to use health care services than patients without these plans. |
| --- | --- |
| Age | Categorical indicator variables for patient ages: 19 to 29, 30 to 39, 40 to 54, and 55 to 64 |
| Gender | Indicator variable=1 if patient is male |
| Concurrent Risk Score | Categorical indicator variables for deciles of patient values in our sample, to allow for flexibility in how patient risk influences spending as patient illness severity increased; BCBSTX calculates the concurrent risk score based on claims in the calendar year of treatment using the Verscend Technologies DxCG concurrent risk score. |
| Wage Index (and Wage Index squared) | Average hourly hospital wage in each local market divided by the national average hourly hospital wage. It is used to construct the geographic adjustment factor (GAF) the Center for Medicare and Medicaid Services uses to adjust fee-for-service reimbursement rates for regional differences in input prices and is updated yearly. |
| Physician Specialty | Categorical indicator variables for physician specialty: family practice, internal medicine, general practice, geriatrics, or hospitalist |
| Practice Size | Categorical indicator variables for the number of patients attributed to each physician group was specified in four categories: practices with up to 500 patients; 501 to 10,000 patients; 10,001 to 15,000 patients, and 15,001+ patients per year. |
| Metropolitan Statistical Area (MSA; also interacted with year) | Categorical indicator variables for the four largest Texas MSAs: Austin, Houston, Dallas, San Antonio |
| Year | Categorical indicator variables for year: 2014, 2015, 2016 |

| **Online Resource 4.**  **Adjusted Effect of Hospital versus Physician Ownership on Median Expenditures** | | | | | |
| --- | --- | --- | --- | --- | --- |
|  | **BCBS** | **BCBS** | **MDPPAS** | **MDPPAS** | **MDPPAS** |
|  |  | *(w/ NPI in MDPPAS)* |  | *(missing TIN name*≠ *physician-owned)* | *(2016 only)* |
| Hospital Owned | 0.0583** | 0.0609** | 0.0666*** | 0.0678*** | 0.0678*** |
|  | (0.0212) | (0.0190) | (0.0181) | (0.0181) | (0.0184) |
|  | [0.0167 - 0.0998] | [0.0237 - 0.0980] | [0.0312 - 0.102] | [0.0323 - 0.103] | [0.0317 - 0.104] |
| Specialty |  |  |  |  |  |
| Family Practice | *Reference* | *reference* | *reference* | *reference* | *reference* |
| Internal Medicine | 0.00423 | -0.00272 | -0.00326 | -0.00240 | 0.00428 |
|  | (0.00994) | (0.00811) | (0.00799) | (0.00811) | (0.00976) |
|  | [-0.0152 - 0.0237] | [-0.0186 - 0.0132] | [-0.0189 - 0.0124] | [-0.0183 - 0.0135] | [-0.0149 - 0.0234] |
| Pediatrics | 0.0727*** | 0.0491 | 0.0433 | 0.0440 | 0.0200 |
|  | (0.0180) | (0.0406) | (0.0449) | (0.0446) | (0.0346) |
|  | [0.0374 - 0.108] | [-0.0305 - 0.129] | [-0.0448 - 0.131] | [-0.0435 - 0.131] | [-0.0479 - 0.0878] |
| General Practice | -0.0374 | -0.0379 | -0.0320 | -0.0277 | -0.0129 |
|  | (0.0541) | (0.0537) | (0.0266) | (0.0274) | (0.0426) |
|  | [-0.143 - 0.0685] | [-0.143 - 0.0674] | [-0.0841 - 0.0202] | [-0.0814 - 0.0260] | [-0.0964 - 0.0705] |
| Geriatrics | --- | --- | 0.0810 | 0.105 | 0.103 |
|  |  |  | (0.0577) | (0.0570) | (0.0901) |
|  |  |  | [-0.0321 - 0.194] | [-0.00696 - 0.216] | [-0.0737 - 0.280] |
| Hospitalist | [not included] | [not included] | 0.0416 | 0.0418 | -0.0117 |
|  |  |  | (0.0343) | (0.0345) | (0.0567) |
|  |  |  | [-0.0256 - 0.109] | [-0.0259 - 0.109] | [-0.123 - 0.0994] |
| Practice Size |  |  |  |  |  |
| Quartile 1 | *Reference* | *reference* | *reference* | *reference* | *reference* |
| Quartile 2 | -0.00660 | 0.0182 | 0.0244 | 0.0247 | 0.0283 |
|  | (0.0195) | (0.0157) | (0.0148) | (0.0148) | (0.0177) |
|  | [-0.0447 - 0.0315] | [-0.0127 - 0.0490] | [-0.00454 - 0.0533] | [-0.00427 - 0.0536] | [-0.00629 - 0.0630] |
| Quartile 3 | 0.00849 | 0.0131 | 0.0188 | 0.0178 | 0.00114 |
|  | (0.0178) | (0.0186) | (0.0182) | (0.0186) | (0.0163) |
|  | [-0.0264 - 0.0434] | [-0.0233 - 0.0496] | [-0.0169 - 0.0544] | [-0.0187 - 0.0543] | [-0.0308 - 0.0331] |
| Quartile 4 | -0.0208 | -0.0196 | -0.0188 | -0.0182 | -0.0224 |
|  | (0.0155) | (0.0145) | (0.0139) | (0.0144) | (0.0157) |
|  | [-0.0511 - 0.00949] | [-0.0480 - 0.00876] | [-0.0461 - 0.00849] | [-0.0465 - 0.0100] | [-0.0532 – 0.00835] |
| Age |  |  |  |  |  |
| 19-29 | 0.0142 | 0.0217*** | 0.0210*** | 0.0221*** | 0.0246** |
|  | (0.00903) | (0.00439) | (0.00442) | (0.00445) | (0.00806) |
|  | [-0.00354 - 0.0319] | [0.0131 - 0.0303] | [0.0123 - 0.0297] | [0.0134 - 0.0308] | [0.00879 – 0.0404] |
| 30-39 | -0.0124** | -0.0128** | -0.0145*** | -0.0139*** | -0.00432 |
|  | (0.00403) | (0.00399) | (0.00397) | (0.00399) | (0.00704) |
|  | [-0.0203 - -0.00450] | [-0.0206 - -0.00501] | [-0.0223 - -0.00671] | [-0.0218 - -0.00610] | [-0.0181 - 0.00947] |
| 40-54 | -0.000166 | -0.00145 | -0.00221 | -0.00265 | 0.00132 |
|  | (0.00319) | (0.00315) | (0.00320) | (0.00322) | (0.00542) |
|  | [-0.00642 - 0.00609] | [-0.00762 - 0.00472] | [-0.00847 - 0.00406] | [-0.00896 - 0.00365] | [-0.00930 - 0.0119] |
| 55-64 | *reference* | *reference* | *reference* | *reference* | *reference* |
| Risk Score |  |  |  |  |  |
| Decile 1 | *reference* | *reference* | *reference* | *reference* | *reference* |
| Decile 2 | 0.503*** | 0.501*** | 0.499*** | 0.500*** | 0.505*** |
|  | (0.00847) | (0.00731) | (0.00727) | (0.00731) | (0.0139) |
|  | [0.486 - 0.519] | [0.487 - 0.515] | [0.485 - 0.513] | [0.485 - 0.514] | [0.478 - 0.533] |
| Decile 3 | 0.869*** | 0.871*** | 0.870*** | 0.869*** | 0.860*** |
|  | (0.0107) | (0.00906) | (0.00909) | (0.00919) | (0.0155) |
|  | [0.849 - 0.890] | [0.853 - 0.889] | [0.853 - 0.888] | [0.851 - 0.887] | [0.829 - 0.890] |
| Decile 4 | 1.178*** | 1.180*** | 1.181*** | 1.180*** | 1.170*** |
|  | (0.0121) | (0.0106) | (0.0107) | (0.0109) | (0.0178) |
|  | [1.155 - 1.202] | [1.160 - 1.201] | [1.160 - 1.202] | [1.159 - 1.201] | [1.135 - 1.204] |
| Decile 5 | 1.453*** | 1.456*** | 1.455*** | 1.454*** | 1.434*** |
|  | (0.0142) | (0.0126) | (0.0126) | (0.0129) | (0.0186) |
|  | [1.425 - 1.481] | [1.431 - 1.480] | [1.431 - 1.480] | [1.428 - 1.479] | [1.397 - 1.470] |
| Decile 6 | 1.729*** | 1.730*** | 1.730*** | 1.728*** | 1.690*** |
|  | (0.0152) | (0.0139) | (0.0141) | (0.0144) | (0.0191) |
|  | [1.699 - 1.759] | [1.703 - 1.757] | [1.702 - 1.757] | [1.700 - 1.756] | [1.653 - 1.728] |
| Decile 7 | 2.045*** | 2.048*** | 2.048*** | 2.047*** | 2.006*** |
|  | (0.0157) | (0.0146) | (0.0146) | (0.0149) | (0.0200) |
|  | [2.014 - 2.076] | [2.019 - 2.076] | [2.019 - 2.076] | [2.018 - 2.076] | [1.967 - 2.045] |
| Decile 8 | 2.418*** | 2.419*** | 2.418*** | 2.417*** | 2.383*** |
|  | (0.0157) | (0.0145) | (0.0145) | (0.0148) | (0.0189) |
|  | [2.388 - 2.449] | [2.390 - 2.447] | [2.390 - 2.447] | [2.388 - 2.446] | [2.346 - 2.420] |
| Decile 9 | 2.952*** | 2.950*** | 2.950*** | 2.948*** | 2.929*** |
|  | (0.0169) | (0.0161) | (0.0160) | (0.0164) | (0.0194) |
|  | [2.919 - 2.985] | [2.919 - 2.982] | [2.918 - 2.981] | [2.916 - 2.980] | [2.891 - 2.967] |
| Decile 10 | 3.827*** | 3.829*** | 3.826*** | 3.826*** | 3.800*** |
|  | (0.0174) | (0.0161) | (0.0156) | (0.0159) | (0.0193) |
|  | [3.793 - 3.861] | [3.797 - 3.860] | [3.796 - 3.857] | [3.795 - 3.857] | [3.762 - 3.838] |
| Male | -0.0272*** | -0.0282*** | -0.0274*** | -0.0277*** | -0.0347*** |
|  | (0.00440) | (0.00447) | (0.00443) | (0.00454) | (0.00567) |
|  | [-0.0358 - -0.0185] | [-0.0369 - -0.0194] | [-0.0361 - -0.0187] | [-0.0366 - -0.0188] | [-0.0459 - -0.0236] |
| Wage Index | -0.733 | 5.108 | -0.487 | -1.747 | 1.422 |
|  | (8.631) | (7.841) | (7.526) | (7.613) | (9.998) |
|  | [-17.65 - 16.18] | [-10.26 - 20.48] | [-15.24 - 14.26] | [-16.67 - 13.17] | [-18.17 - 21.02] |
| Wage Index squared | 0.636 | -2.724 | 0.335 | 1.027 | -0.547 |
|  | (4.895) | (4.441) | (4.250) | (4.299) | (5.676) |
|  | [-8.958 - 10.23] | [-11.43 - 5.979] | [-7.995 - 8.665] | [-7.399 - 9.453] | [-11.67 - 10.58] |
| Consumer Driven Health Plan | -0.00834 | -0.00964* | -0.00920* | -0.00975* | -0.00831 |
|  | (0.00440) | (0.00404) | (0.00393) | (0.00398) | (0.00635) |
|  | [-0.0170 - 0.000290] | [-0.0176 - -0.00172] | [-0.0169 - -0.00149] | [-0.0176 - -0.00195] | [-0.0208 - 0.00414] |
| Year |  |  |  |  |  |
| 2014 | *Reference* | *reference* | *reference* | *reference* | --- |
| 2015 | 0.0680** | 0.0486* | 0.0577** | 0.0597** | --- |
|  | (0.0213) | (0.0192) | (0.0184) | (0.0188) |  |
|  | [0.0262 - 0.110] | [0.0110 - 0.0863] | [0.0217 - 0.0937] | [0.0229 - 0.0965] |  |
| 2016 | 0.0811** | 0.0603* | 0.0718** | 0.0731** | --- |
|  | (0.0273) | (0.0249) | (0.0240) | (0.0246) |  |
|  | [0.0276 - 0.135] | [0.0115 - 0.109] | [0.0247 - 0.119] | [0.0249 - 0.121] |  |
| Metro Area-Year Interaction |  |  |  |  |  |
| San Antonio x 2014 | -0.0577 | -0.105 | -0.0911 | -0.0880 | --- |
|  | (0.0641) | (0.0553) | (0.0523) | (0.0532) |  |
|  | [-0.183 - 0.0679] | [-0.213 - 0.00370] | [-0.194 - 0.0114] | [-0.192 - 0.0162] |  |
| San Antonio x 2015 | -0.0509 | -0.0802* | -0.0752 | -0.0736 | --- |
|  | (0.0468) | (0.0403) | (0.0385) | (0.0390) |  |
|  | [-0.143 - 0.0407] | [-0.159 - -0.00119] | [-0.151 - 0.000156] | [-0.150 - 0.00286] |  |
| San Antonio x 2016 | -0.0633 | -0.0906* | -0.0882* | -0.0879* | -0.0627 |
|  | (0.0441) | (0.0392) | (0.0377) | (0.0381) | (0.0494) |
|  | [-0.150 - 0.0231] | [-0.167 - -0.0138] | [-0.162 - -0.0144] | [-0.163 - -0.0132] | [-0.160 - 0.0341] |
| Dallas-Fort Worth x 2014 | 0.0744* | 0.0678* | 0.0778** | 0.0770** | --- |
|  | (0.0292) | (0.0297) | (0.0283) | (0.0293) |  |
|  | [0.0171 - 0.132] | [0.00967 - 0.126] | [0.0223 - 0.133] | [0.0195 - 0.135] |  |
| Dallas-Fort Worth x 2015 | 0.0503* | 0.0517* | 0.0568** | 0.0563** | --- |
|  | (0.0203) | (0.0208) | (0.0196) | (0.0203) |  |
|  | [0.0106 - 0.0900] | [0.0110 - 0.0924] | [0.0183 - 0.0953] | [0.0165 - 0.0960] |  |
| Dallas-Fort Worth x 2016 | 0.0319 | 0.0501** | 0.0477** | 0.0453** | 0.0494** |
|  | (0.0198) | (0.0157) | (0.0148) | (0.0151) | (0.0161) |
|  | [-0.00681 - 0.0706] | [0.0194 - 0.0808] | [0.0187 - 0.0766] | [0.0157 - 0.0748] | [0.0179 - 0.0810] |
| Austin x 2014 | -0.00430 | -0.0245 | -0.0106 | -0.00681 | --- |
|  | (0.0439) | (0.0378) | (0.0362) | (0.0370) |  |
|  | [-0.0904 - 0.0818] | [-0.0986 - 0.0495] | [-0.0815 - 0.0603] | [-0.0794 - 0.0658] |  |
| Austin x 2015 | -0.00436 | -0.00521 | -0.000642 | 0.00121 | --- |
|  | (0.0307) | (0.0251) | (0.0243) | (0.0247) |  |
|  | [-0.0646 - 0.0559] | [-0.0545 - 0.0441] | [-0.0483 - 0.0470] | [-0.0472 - 0.0496] |  |
| Austin x 2016 | 0.00741 | 0.0126 | 0.0133 | 0.0140 | 0.0190 |
|  | (0.0236) | (0.0184) | (0.0179) | (0.0182) | (0.0209) |
|  | [-0.0388 - 0.0537] | [-0.0235 - 0.0487] | [-0.0217 - 0.0483] | [-0.0217 - 0.0497] | [-0.0219 - 0.0600] |
| Houston x 2014 | *Reference* | *reference* | *reference* | *reference* | --- |
| Houston x 2015 | *Reference* | *reference* | *reference* | *reference* | --- |
| Houston x 2016 | *Reference* | *reference* | *reference* | *reference* | *reference* |
| Constant | 6.165 | 3.663 | 6.201 | 6.771* | 5.272 |
|  | (3.754) | (3.419) | (3.293) | (3.332) | (4.372) |
|  | [-1.191 - 13.52] | [-3.037 - 10.36] | [-0.252 - 12.65] | [0.242 - 13.30] | [-3.297 - 13.84] |
|  |  |  |  |  |  |
| Observations | 1,531,120 | 1,410,504 | 1,365,477 | 1,329,200 | 443,366 |
| Robust SE in parentheses and CI in brackets | | | | | |
| *** p<0.001, ** p<0.01, *p<0.05 | | |  | | |

**Online Resource 5.**

**Sensitivity Analysis: Adjusted Effect of Hospital versus Physician Ownership on Median Expenditures by Specialty^a^**

|  | **Internal Medicine** | | **Family Practice** | |
| --- | --- | --- | --- | --- |
|  | **BCBS** | **MDPPAS** | **BCBS** | **MDPPAS** |
|  |  |  |  |  |
| Hospital Owned | 0.0666* | 0.0991*** | 0.0547* | 0.0519** |
|  | (0.0271) | (0.0217) | (0.0212) | (0.0185) |
|  | (0.0135 - 0.120) | (0.0566 - 0.142) | (0.0130 - 0.0963) | (0.0157 - 0.0881) |
|  |  |  |  |  |
| Observations | 418,053 | 394,243 | 1,094,209 | 939,247 |
| Robust SE in parentheses and CI in brackets  *** p<0.001, ** p<0.01, * p<0.05  **^a^** The estimates are adjusted for practice size, year, age, gender, concurrent risk score, participation in a consumer-directed health plan, MSA, and physician specialty. The year indicator variables capture increases in spending resulting from inflation. | | | | |
